# First record of the genus *Boholina* (Copepoda, Calanoida, Pseudocyclopidae) in Vietnam, with description of a new species from an anchialine cave in Tra Ban Island

**DOI:** 10.3897/zookeys.977.55040

**Published:** 2020-10-22

**Authors:** Duc Luong Tran, Cheon Young Chang

**Affiliations:** 1 Department of Aquatic Ecology, Institute of Ecology and Biological Resources, Academy of Science and Technology, 18, Hoang Quoc Viet Rd, Caugiay District, Hanoi, Vietnam Institute of Ecology and Biological Resources, Academy of Science and Technology Hanoi Vietnam; 2 Graduate University of Science and Technology, Vietnam Academy of Science and Technology, 18 Hoang Quoc Viet Rd, Cau Giay District, Hanoi, Vietnam Graduate University of Science and Technology, Vietnam Academy of Science and Technology Hanoi Vietnam; 3 Department of Biological Science, Daegu University, Gyeongsan 712-714, South Korea Daegu University Gyeongsan South Korea

**Keywords:** Bai Tu Long Bay, Biodiversity, new species, SEM, taxonomy

## Abstract

A new species, *Boholina reducta***sp. nov.**, was found in a brackish pool within an anchialine cave in Tra Ban Island in Bai Tu Long Bay, north Vietnam. The new species is clearly distinguished from all the six species currently known in the genus *Boholina* by the following unique characteristics: reduction of the septum between gonopores; narrow and pointed rostrum; basal segment of mandibular palp armed with three setae; maxillule without seta on the basal exite, and exopod with 11 setae; second and third endopodal segments of the maxilliped bearing three setae each; exopod of male right leg 5 2-segmented, with two strong and one vestigial spines on the outer margin of the distal segment; and last exopodal segment of female leg 5 bearing only one spine on the outer margin. We provide a description of the new species, along with detailed illustrations and scanning electron microscopy photographs. The identification key to *Boholina* species is updated as well. This is the first record of the genus *Boholina* from Vietnam.

## Introduction

The family Boholinidae was established for a single genus *Boholina* by Fosshagen and Iliffe (1989) on the basis of a combination of morphological characteristics: well-developed mouthparts; 3-segmented rami on P1–P4; female P5 with 3-segmented exopod and slightly reduced 2-segmented endopod; right antennule of male geniculated; and male P5 with a highly complex grasping organ. Fosshagen and Iliffe (1989) argued that it differed from the families Pseudocyclopidae Giesbrecht, 1893 and Ridgewayiidae Wilson, 1958 by the modified terminal spine on the distal exopodal segment of P4 and the inner seta on the coxal segment of female P5 (Fosshagen and Iliffe 1989).

However, Bradford-Grieve et al. (2014) considered Boholinidae and Ridgewayiidae as junior synonyms of Pseudocyclopidae, based on a morphology-based cladistic analysis, and placed the genus *Boholina* in the Pseudocyclopidae. To date, 14 genera have been recognized in Pseudocyclopidae as follows: *Badijella* Kršinic, 2005; *Boholina* Fosshagen, 1989; *Brattstromia* Fosshagen, 1991; *Exumella* Fosshagen, 1970; *Exumellina* Fosshagen, 1998; *Hondurella* Suárez-Morales & Iliffe, 2007; *Normancavia* Fosshagen & Iliffe, 2003; *Pinkertonius* Bradford-Grieve, Boxshall & Blanco-Bercial, 2014; *Placocalanus* Fosshagen, 1970; *Pseudocyclops* Brady, 1872; *Ridgewayia* Thompson & Scott, 1903; *Robpalmeria* Fosshagen & Iliffe, 2003; *Stargatia* Fosshagen & Iliffe, 2003 and *Stygoridgewayia* Tang, Barron & Goater, 2008 (Walter and Boxshall 2020). Among these genera, *Pseudocyclops* and *Ridgewayia* have a worldwide distribution from temperate, subtropical to tropical shallow waters (Ohtsuka et al. 1999; Boxshall and Halsey 2004; Figueroa 2011a, b). Other genera are known from the North Atlantic and Mediterranean (*Exumella*, *Badijella*), Belize (*Brattstromia*), Bahamas (*Exumellina*, *Normancavia*, *Robpalmeria*, *Stargatia*), Western Caribbean (*Hondurella*), Australia and New Zealand (*Stygoridgewayia*, *Pinkertonius*). Most species of the Pseudocyclopidae were reported from shallow benthopelagic or anchialine cave habitats, while *Stygoridgewayia* was found in fresh groundwater in Australia (Bradford-Grieve et al. 2014).

The genus *Boholina* currently comprises six valid species: *B. crassicephala* Fosshagen & Iliffe, 1989 and *B. purgata* Fosshagen & Iliffe, 1989 from an anchialine cave on San Vicente, Bohol Island (Philippines); *B. parapurgata* Boxshall & Jaume, 2012 and *B. munaensis* Boxshall & Jaume, 2012 from anchialine and brackish waters of low salinity in Muna Island (Indonesia); *B. ganghwaensis* Moon & Soh, 2014 from burrows of the manicure crab in muddy habitats on Ganghwa Island, Korea; and the recently introduced *B. laorsriae* Boonyanusith, Wongkamhaeng & Athibai, 2020 from a freshwater pool within a cave located about 6.5 km from the Andaman Sea, Thailand (Fosshagen and Iliffe 1989; Boxshall and Jaume 2012; Moon and Soh 2014; Boonyanusith et al. 2020).

In this paper, we describe a new species of *Boholina*, based on specimens from an anchialine habitat of a karstic cave in Tra Ban Island, north Vietnam, along with detailed illustrations drawn under a differential interference microscope and by scanning electron microscopy. We also discuss its morphological relationships with congeners.

## Materials and methods

**Figure 1.**
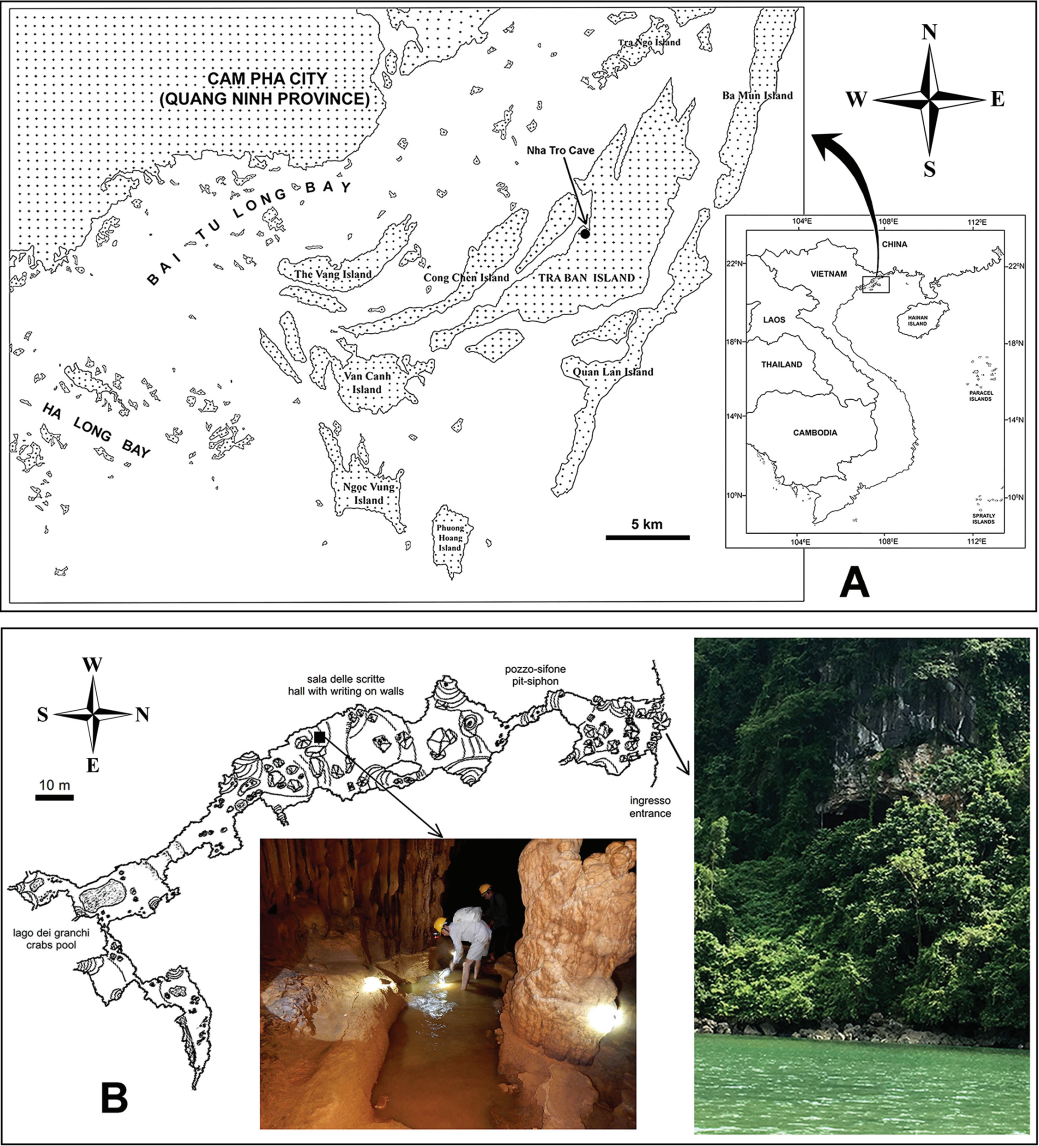
Sampling locations of *Boholina reducta* sp. nov. **A** map of the Bai Tu Long Bay showing the location of Nha Tro Cave (arrow) **B** map of the Nha Tro Cave (cited from Donatis et al. 2010), designating the entrance from the sea and the type locality of the new species.

Nha Tro Cave (or Hang Cam Cave) is located in Vietnam, on Tra Ban Island in Bai Tu Long Bay. The island is 20 km from Cam Pha City in Quang Ninh Province, and about 12 km from the mainland; it has an area of about 76.37 km² (Fig. 1A). The main geological composition of the island is stratified limestone, silicate and claystone (Uong et al. 2013).

The cave has a large entrance at 17 m above sea level (20°57'31.0"N, 107°29'12.1"E); a few meters from the entrance there is a larger downward-sloping hall (Fig. 1B). On the left of the cave is a steeply climbing branch, terminating after a few meters. On the right is the main gallery along the length of the cave with enormous boulders due to rockfalls and large concretions.

The floor is composed of pools and clay deposits; concretions are abundant with several discs, the largest one reaching three meters in diameter. The dimension of the cave is about 350 m in length, 10 m high and 15 m in depth (Donatis et al. 2010).

Copepods were collected from a pool inside the Nha Tro Cave (Fig. 1B). The pool is in a permanently dark section about 200 m from the cave exit. Physico-chemical characteristics of the pool on 9 May 2018 are as follows: water temperature 19.8 °C; pH 7.82; dissolved oxygen 0.76 mg/L; water hardness (CaCO_3_) 154 mg/L; electrical conductivity 1.12 mS/cm; salinity 0.18‰. Copepods were taken from the pool in knee-deep water, with a hand net with mesh size of 80 µm. They were fixed in about 80% ethanol in the field, and later stored in about 70% ethanol. Specimens were dissected and mounted in glycerol or lactophenol. The mounted specimens were observed under a differential interference contrast microscope with Nomarski optics (Nikon Eclipse Ni-U). All drawings were made with the aid of a camera lucida.

Material used for scanning electron microscopy (SEM) was fixed in 2.5% glutaraldehyde in 0.1M phosphate buffer (pH 7.2–7.4) for 2 hours, followed by fixation in 1% cold osmium tetroxide (at about 5°C) in the same buffer for 12 hours. After dehydration through a graded series of ethanol (70, 80, 90, 95 and 100%) for 30 minutes each, the material was critical point dried, coated with gold/palladium, and then examined with a scanning electron microscope Hitachi TM3000 TableTop operated at 15 KV. The following abbreviations are used, where required, throughout the text and figures: Endp = endopod; Exp = exopod; P1–P5 = swimming legs 1–5. General terminology for the description follows Huys and Boxshall (1991), including analysis of caudal setae (I–VII) and antennule segmentation (evident segments labeled with Arabic numerals, and ancestral segments with Roman numerals), and the terminology and homology for maxillary and maxilliped structures by Ferrari and Ivanenko (2001, 2008) is adopted herein.

Type specimens are deposited in the Institute of Ecology and Biological Resources (IEBR), Hanoi, Vietnam.

## Taxonomy

### Order Calanoida G.O. Sars, 1903

**Family Pseudocyclopidae Giesbrecht, 1893**

**Genus *Boholina* Fosshagen & Iliffe, 1989**

#### Boholina reducta sp. nov.

Taxon classificationAnimalia

A046CE43-5CFF-5A76-9AE9-C525E0BE4F31

http://zoobank.org/909D70A1-A05C-4E8B-B222-077DE414F090

Figs 2 , 3 , 4 , 5 , 6 , 7 , 8 , 9

##### Type material.

***Holotype***: ♀ (IEBR-COP3480–3481), 933 µm long; Quang Ninh Province, Tra Ban Island, Nha Tro Cave; 20°57'31.0"N, 107°29'12.1"E; 9 May 2018; D.L. Tran leg.; a pool inside the cave; dissected and mounted on two slides in glycerol. ***Allotype***: ♂ (IEBR-COP3482–3483), 812 µm long; same data as for holotype; dissected and mounted on two slides in glycerol. ***Paratypes***: 2 ♂♂ (IEBR-COP3484, 3485) and 5 ♀♀ (IEBR-COP3486–3490); same data as for holotype; dissected and mounted in glycerol.

##### Additional material.

60 ♂♂ and more than 100 ♀♀, same data as for holotype, preserved in 70% ethanol, IEBR-COP-AED05.2018.13; 5 ♂♂ and 5 ♀♀, same data as for holotype, prepared for SEM examination, retained in the collection of the first author (DLT).

##### Type locality.

A pool inside the Nha Tro Cave (geographic coordinates of the cave entrance: 20°57'31.0"N, 107°29'12.1"E) in Tra Ban Island, Bai Tu Long Bay, Quang Ninh Province, north Vietnam.

##### Etymology.

The proposed name refers to reduction of the terminal spine on the distal exopod segment of P5 in the male as well as the proximal outer spine on the distal exopodal segment of P5 in the female, which are the most remarkable characteristics differentiating this new species from all the congeneric species of *Boholina*.

##### Diagnosis.

Boholinid form in both sexes. Postero-lateral corners of second and third pedigerous somites rounded, fourth and fifth pedigerous somites completely fused. Rostrum represented by a narrow chitinized projection with pointed tip. Medial lobe of distal segment A2 endopod with nine setae. Mandibular palp basis with three setae; distal segment of endopod with 11 setae; seta on first segment of exopod present. Maxillule exopod with 11 setae and seta on basal exite absent. Second and third segments of maxilliped endopod with three setae each. Terminal spine on exopod of leg 4 modified with row of large spinules on mid-inner margin. ***Female***: Gonopores on double-somite located close together on mid-ventral surface, septum between gonopores reduced to vestige deep inside genital opening. P5 Exp-3 with only one spine on outer margin and four setae on inner margin; distal segment of P5 endopod with one seta on outer margin. ***Male***: Process at antepenultimate segment of right antennule absent. Right P5 exopod 2-segmented; distal segment with three spines, including a vestigial one on outer margin, while terminal and inner spine absent.

##### Description of adult female.

**Figure 2.**
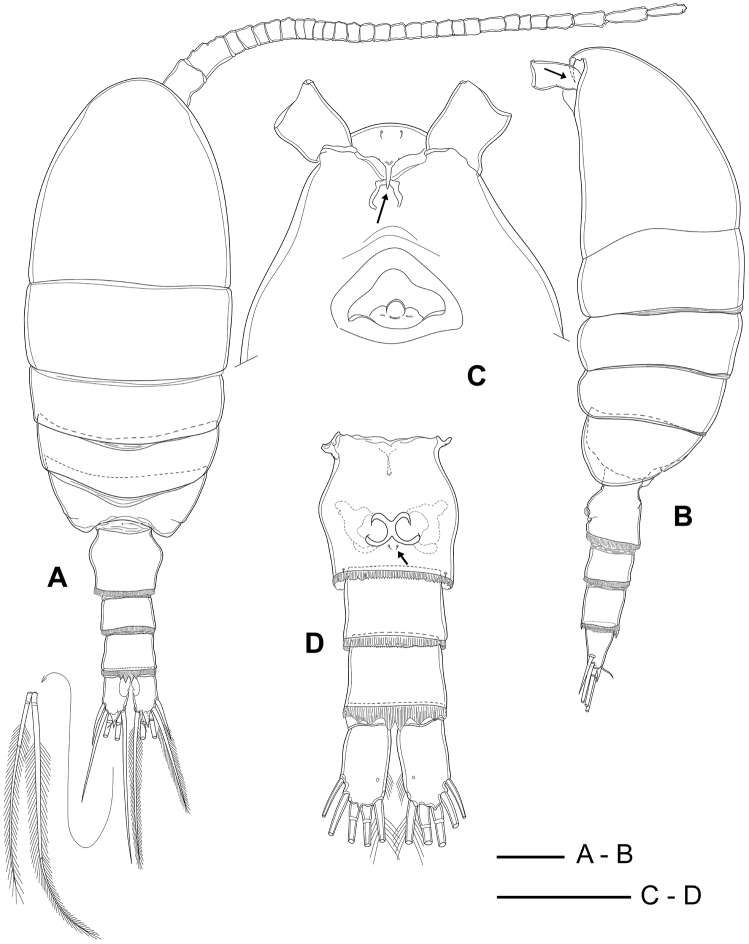
*Boholina reducta* sp. nov., holotype female. **A** habitus, dorsal view **B** habitus, lateral view (arrow indicating tip of rostrum) **C** rostrum, ventral view (arrow indicating tip of rostrum) **D** urosome, ventral view (arrow indicating sensilla near gonopores). Scale bars: 100 µm.

Total length (without furcal setae) 858–944 µm (mean 892 µm, *N* = 20). Ratio of prosome to urosome length about 3.1:1 (Fig. 2A, B). Prosome ovoid in dorsal view, 5-segmented, comprising cephalosome; first pedigerous somite separated from cephalosome; second and third free pedigerous somites with postero-lateral corners rounded; fourth and fifth pedigerous somites completely fused.

Urosome 4-segmented, comprising genital double-somite, two free abdominal somites and anal somite. Genital double-somite slightly asymmetrical, widest about at mid-length; posterior margin ornamented with smooth hyaline membrane dorsally and small dentate hyaline frill ventrally, about as long as wide; paired gonopores equal in size, located close together on mid-ventral surface, the septum between gonopores reduced to vestige, deep inside genital opening; gonoporal plates small, and gonoporal slits large; two pairs of sensilla present (Figs 2D, 9C, D, arrows), one pair positioned adjacent to posterior margin of gonopores and second pair located ventrolaterally near posterior margin of double-somite. Third and fourth abdominal somites cylindrical, subequal in length (Fig. 2D); third with finely serrated hyaline membrane all around posterior margin, fourth with posterior margin hyaline membrane expanded mid-dorsally to four large spines functioning as pseudoperculum concealing anal opening and mid-ventrally with finely serrated hyaline membrane on posterior margin. Anal somite extremely short, posterior margin smooth, concealed within posterior rim and hyaline membrane of second free abdominal somite.

Caudal rami (Fig. 2A, D) short, about 1.5 times longer than wide, with pointed dorsal process in middle of distal margin; distal inner margin with a row of setules; ventral surface with a small pore near inner distal edge; ornamented six caudal setae; seta I lacking, seta II spiniform, about 1.2 time as long as caudal ramus; setae III–VI plumose, ratio of setae V:IV:VI:III:II as 5.8:4.3:4.1:2.4:1.0; dorsal seta VII short, naked, about 0.5 times as long as seta II.

Rostral filaments absent, rostrum represented by a narrow chitinized projection with pointed tip (Figs 2B, C, 9B, arrows); pair of long sensilla present in proximal part of rostrum.

**Figure 3.**
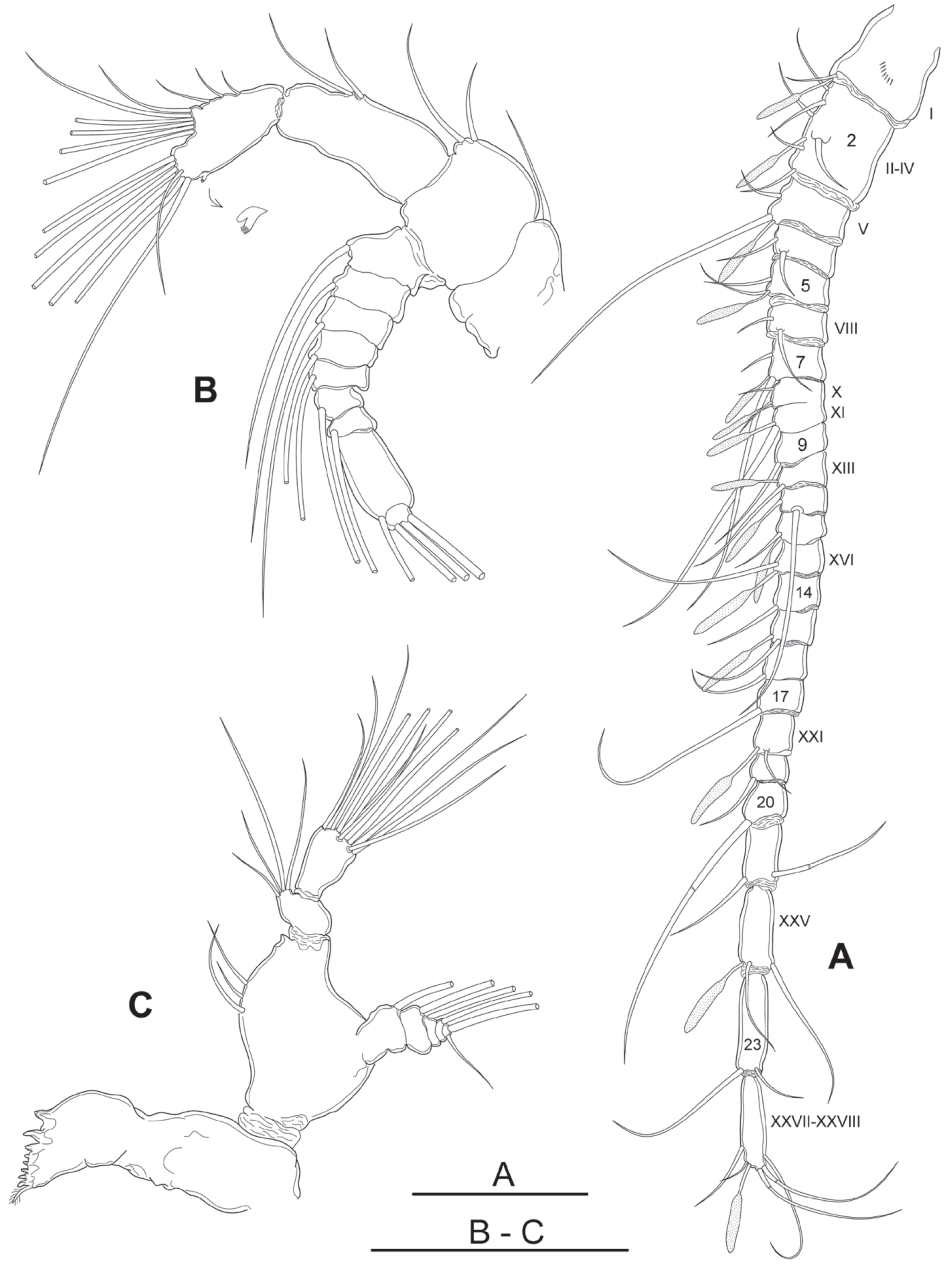
*Boholina reducta* sp. nov., holotype female. **A** antennule **B** antenna (arrow indicating small serrated process) **C** mandible. Scale bars: 100 µm.

Antennules (Figs 2A, 3A) symmetrical, extending to middle area of pedigerous somite 5, 24-segmented with ancestral segments II–IV and XXVII–XXVIII fused, X–XI party fused, other articulations expressed, ventral surface of segment 1 with a row of small oblique spines. Armature formula as follows (s - setae, ae - aesthetasc): segment 1 (ancestral segment I) 1s + 1ae, segment 2 (II–IV) 6s + 1ae, segment 3 (V) 2s + 1ae, segment 4 (VI) 2s, segment 5 (VII) 2s + 1ae, segment 6 (VIII) 2s, segment 7 (IX) 2s + 1ae, segment 8 (X–XI) 3s + 2ae, segment 9 (XII) 1s, segment 10 (XIII) 1s + 1ae, segment 11 (XIV) 1s + 1ae, segment 12 (XV) 1s + 1ae, segment 13 (XVI) 1s + 1ae, segment 14 (XVII) 1s, segment 15 (XVIII) 1s + 1ae, segment 16 (XIX) 1s, segment 17 (XX) 1s, segment 18 (XXI) 1s + 1ae, segment 19 (XXII) 1s, segment 20 (XXIII) 1s, segment 21 (XXIV) 2s, segment 22 (XXV) 2s + 1ae, segment 23 (XXVI) 2s, segment 24 (XXVII–XXVIII) 5s + 1ae.

Antenna (Figs 3B, 8A) biramous. Coxa and basis separate, coxa small, with a seta. Basis robust with two setae on inner distal corner. Endopod 2-segmented; proximal segment elongated, 2.2 times as long as wide, with two naked setae at 1/3 distal length of inner margin; distal segment with two lobes, medial lobe bearing six setae distally and three setae on inner distal margin, outer lobe with six long setae terminally and a short sub-terminal seta, outer margin ornamented with small serrated process (Fig. 3B, arrow) subdistally on medial margin and adjacent tiny spinules. Exopod 9-segmented, with setal formula of 1, 1, 1, 1, 1, 1, 1, 1, 3.

Mandible (Figs 3C, 8B) with about eight small teeth on gnathobase plus small distal spinulose seta; ventral-most teeth largest. Mandibular palp biramous; basis robust with three unequal smooth setae on inner margin. Exopod 5-segmented, setal formula 1, 1, 1, 1, 2. Endopod 2-segmented, proximal with four smooth setae at distomedial angle, distal segment with 11 naked setae on distal margin.

**Figure 4.**
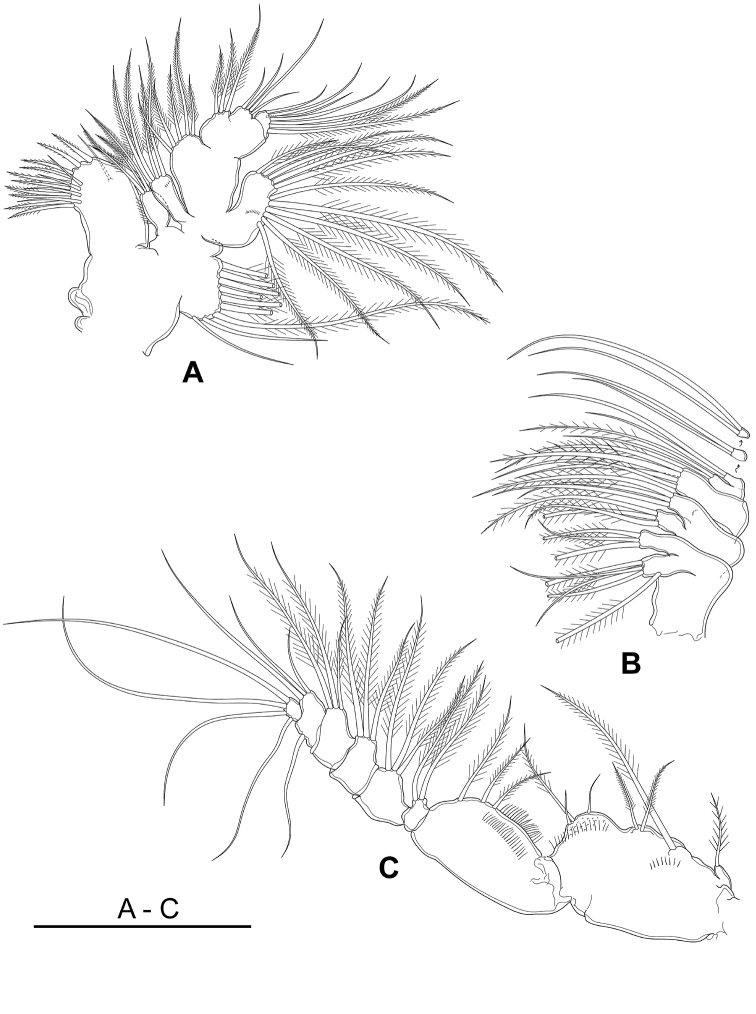
*Boholina reducta* sp. nov., holotype female. **A** maxillula **B** maxilla **C** maxilliped. Scale bar: 100 µm.

Maxillule (Figs 4A, 8C) with 10 marginal spinulose spines, one naked seta on anterior surface and four stiff setae on posterior surface of praecoxal arthrite. Coxal epipodite with seven plumose setae and two naked setae; coxal endite with four plumose setae. Basis fused to exopod, proximal basal endite with four plumose setae, distal basal endite sub-separated with endopod carrying five plumose setae; basal exite bared. Exopod completely fused to basis bearing 11 plumose setae, posterior surface with a slender oblique row of setules. Endopod with segments 1 and 2 fused, segments 2 and 3 separate, with three plumose, four bare and seven (2 plumose + 5 bare) setae, respectively.

Maxilla (Figs 4B, 8D) 7-segmented, comprising syncoxa, basis and 5-segmented endopod. Syncoxa with five setae on praecoxal endite and three setae on coxal endite. Basis with two endites, each armed with three apical setae. Endopod 5-segmented; proximal endopodal segment developed, enditic-like; second and third segments partly fused; other segments divided; setal formula 4, 2, 2, 2, 2.

Maxilliped (Figs 4C, 8E) well developed, 8-segmented with syncoxa, basis and free 6-segmented endopod. Syncoxa comprising praecoxa and coxa, completely fused; praecoxa with three endites; proximal and middle praecoxal endites each bearing one plumose seta, distal praecoxal endite with two plumose setae; coxa with one endite armed with one long plumose seta and two short, naked setae; oblique rows of spinules situated on posterior proximal of middle praecoxal endite and coxal endite. Basis elongated, armed with three plumose setae, inserted at distal 1/3 of medial margin and carrying rows of setules along medial margin and posterior face of segment. Endopod 6-segmented, with setal formula 2, 3, 3, 3, 3 + 1, 4.

**Figure 5.**
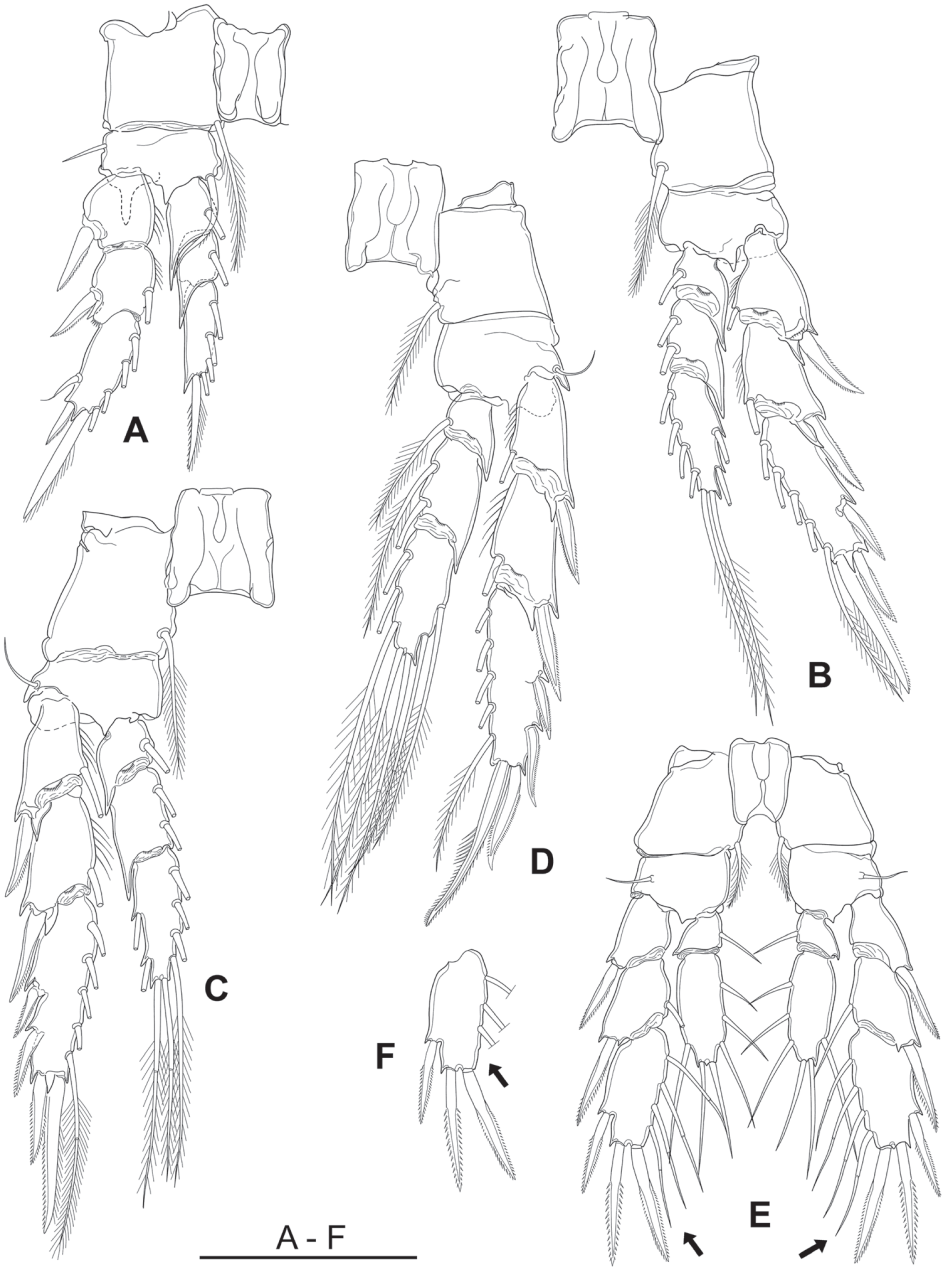
*Boholina reducta* sp. nov. **A**–**E** holotype female, **F** paratype female. **A** P1, posterior **B** P2, posterior **C** P3, posterior **D** P4, posterior **E** P5, posterior (arrows indicating the presence of distal inner setae) **F** distal segment exopod of left P5 (arrow indicating a trace of the distal inner seta). Scale bar: 100 µm.

P1–P4 (Fig. 5A–D) biramous, with 3-segmented rami. Intercoxal sclerites of P1–P4 naked on both frontal and caudal surfaces. First to second endopodal segments of P1–P4 with pointed process on distolateral corners. Articulations between endopodal and exopodal segments ornamented with rows of tiny spinules. Armature of P1–P5 as in Table 1.

**Table 1.** Armature of female P1–P5 in *B. reducta* sp. nov. (spines denoted by Roman, and setae by Arabic numerals). Armature on the lateral margin of any segment is given ﬁrst, followed by the elements on the apical and medial margins.

	Coxa	Basis	Exopodite	Endopodite
P1	0-1	1-1	I-0; I-1; II,I,4	0-1; 0-1; 0,I+1,3
P2	0-1	0-0	I-1; I-1; II,I,5	0-1; 0-2; 2,2,4
P3	0-1	1-0	I-1; I-1; III,I,5	0-1; 0-2; 2,2,4
P4	0-1	1-0	I-1; I-1; III,I,5	0-1; 0-2; 2,2,3
P5	0-1	1-0	I-0; I-1; I,II,4	0-1; 1,2,3

Basis of P1 with distally pointed digitiform process on anterior; inner basal seta crooked, bilaterally spinulate, reaching to distal end of second endopodal segment; second exopodal segment with conspicuous spinulate process distally in outer distal corner of segment. Outer proximal spine on third exopodal segment of P1 flagellate, other outer spines on P2–P4 with serrate marginal membrane(s) as figured. Terminal spine on exopod of P1 with naked outer margin and plumose internally; on P2 and P3 with serrate membrane externally and plumose internally; that on P4 modified with row of large spinules on mid-inner margin and armed slender spinules on outer margin and distal part of inner margin (Figs 5D, 9A).

P5 (Figs 5E, F, 8F) biramous, with 3-segmented exopod and 2-segmented endopod, intercoxal sclerite smooth and unornamented. Basis small, 1.4 times as wide as long, with acute process on posterior surface near base of exopod. Exopod longer than endopod: tip of endopod only reaching to proximal inner seta on third exopodal segment. Distal endopodal segment 2.4 times as long as wide, armed with three inner, two apical and one outer naked setae. First and second exopodal segments each ornamented with a small pore on anterior surface at origin of outer spine. Distal exopodal segment 2.1 times as long as wide, bearing lateral spine (about 38–41 µm), subapical and apical spines of same length (about 51–54 µm); inner margin with four naked setae.

##### Description of adult male.

**Figure 6.**
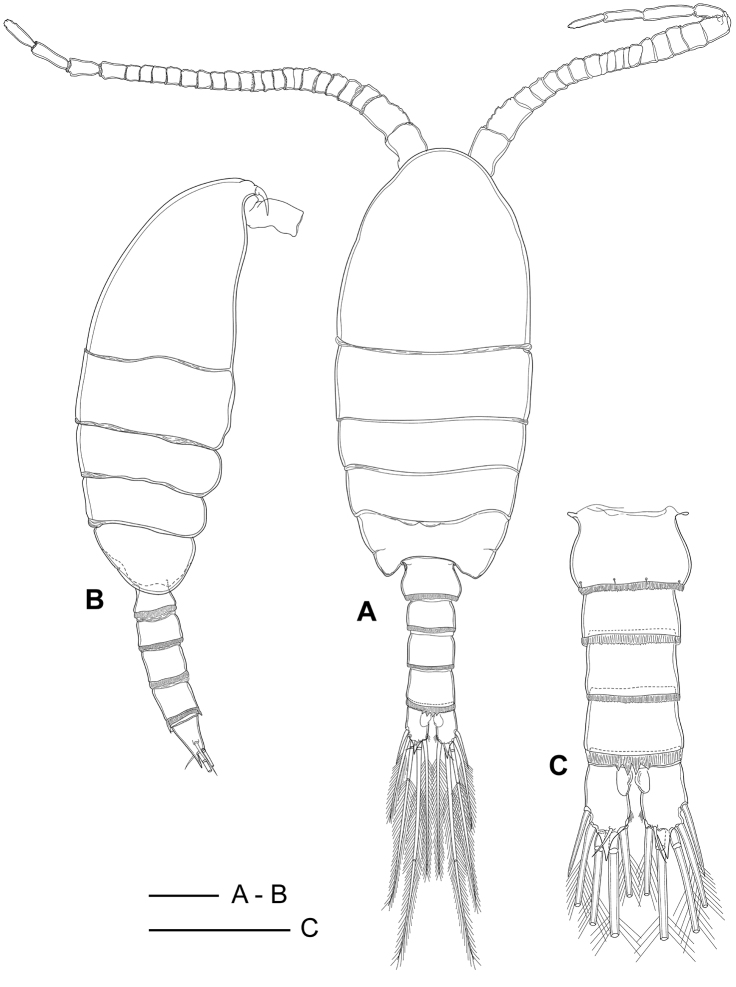
*Boholina reducta* sp. nov., allotype male. **A** habitus, dorsal view **B** habitus, lateral view **C** urosome, dorsal view. Scale bars: 100 µm.

Body smaller than female, 756–825 µm long (mean 799 µm, *N* = 20). Ratio of prosome to urosome length about 2.7:1 (Fig. 6A, B). Prosome 5-segmented as in female: cephalosome completely separated from ﬁrst pedigerous somite; second and third pedigerous somites with rounded ventroposterior corners; fourth and fifth pedigerous somites fused, with only marked articulation dorsolaterally; posterior corners of fifth pedigerous somites rounded in lateral view.

Urosome 5-segmented (Fig. 6A–C), comprising genital somite, three free abdominal somites and anal somite. Genital somite slightly asymmetrical, distal part of right margin protuberant, slightly more expanded than left margin (Fig. 6C); both lateral margins smooth; four sensilla along dorsoposterior margin; posterior margin with finely serrated hyaline membrane. Second to fourth free abdominal somites cylindrical, subequal in size; second and third somites with finely serrated hyaline membrane on posterior margin; fourth with posterior margin hyaline membrane expanded mid-dorsally to four large spines functioning as pseudoperculum concealing anal opening and mid-ventrally with finely serrated hyaline membrane on posterior margin. Anal somite short, ring-like, with posterior margin smooth.

Caudal rami symmetrical, 1.5–1.6 times as long as wide (mean 1.57, *N* = 10), bearing distal spinous process dorsally and row of small setules on distal inner margin; ventral surface with a small pore near inner distal edge; ornamented six caudal setae, included to caudal setae II–VII and absent seta I.

**Figure 7.**
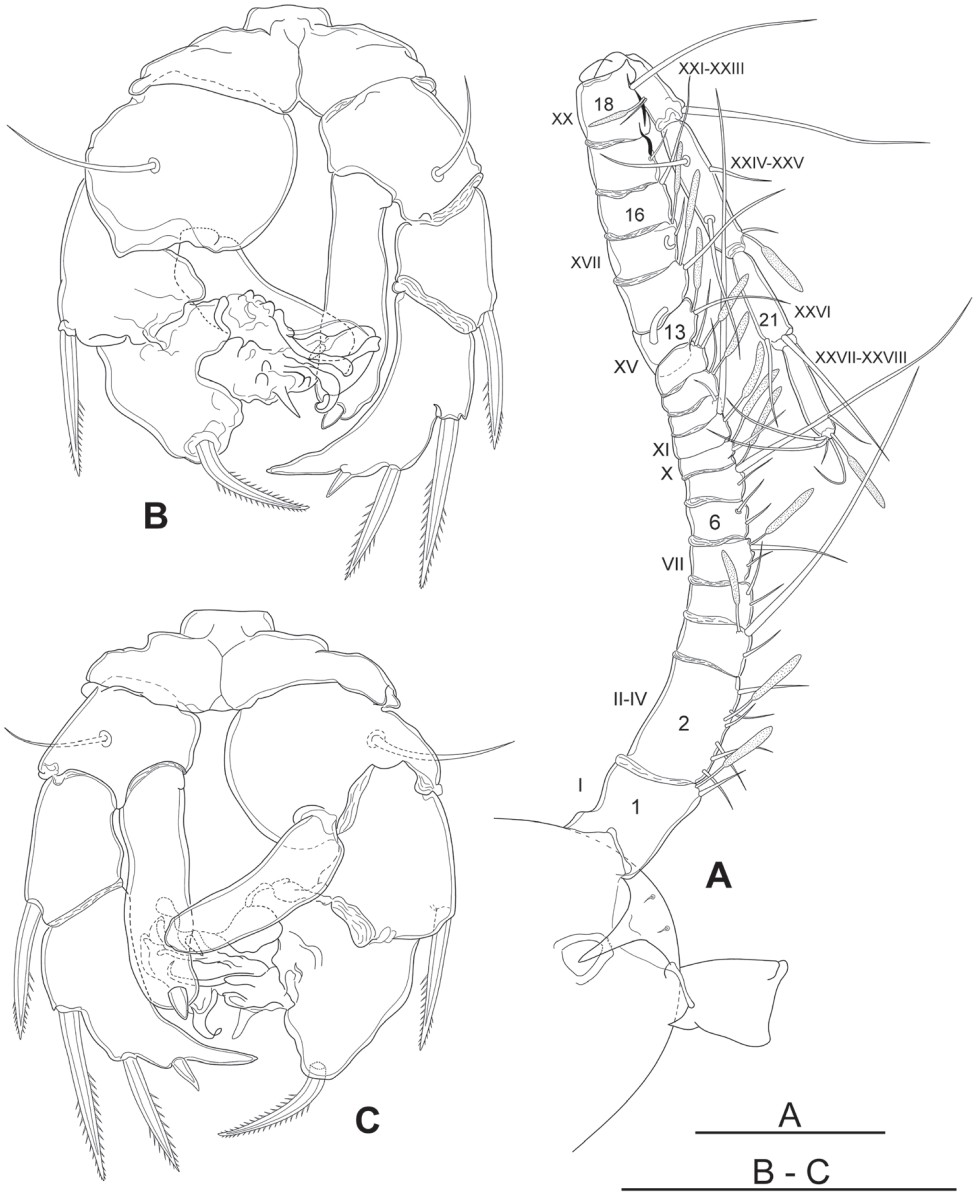
*Boholina reducta* sp. nov., allotype male. **A** rostrum and antennule, ventral view **B** P5, posterior **C** P5, anterior. Scale bars: 100 µm.

Antennules shorter than in female, asymmetrical. Left antennule non-geniculate, 24-segmented and extending to middle area of pedigerous somite 5, armature segments as in female. Right antennule (Fig. 7A) geniculate, 22-segmented, extending to middle of last pedigerous somite; segments 13–18 broadened; segments 17–18 with knife-like projection on inner margins; armature formula as follows (s - setae, ae - aesthetasc): segment 1 (ancestral segment I) 1s + 1ae, segment 2 (II–IV) 6s + 1ae; segment 3 (V) 2s + 1ae; segment 4 (VI) 2s; segment 5 (VII) 2s + 1ae; segment 6 (VIII) 2s; segment 7 (IX) 2s + 1ae; segment 8 (X) 1s + 1ae; segment 9 (XI) 1s + 1ae; segment 10 (XII) 1s; segment 11 (XIII) 1s + 1ae; segment 12 (XIV) 1s + 1ae; segment 13 (XV) 1s + 1ae; segment 14 (XVI) 1s + 1ae; segment 15 (XVII) 1s + 1ae; segment 16 (XVIII) 1s; segment 17 (XIX) 1s; segment 18 (XX) 1s; segment 19 (XXI–XXIII) 2s + 1ae; segment 20 (XXIV–XXV) 4s + 1ae; segment 21 (XXVI) 2s; segment 22 (XXVII–XXVIII) 5s + 1ae.

**Figure 8.**
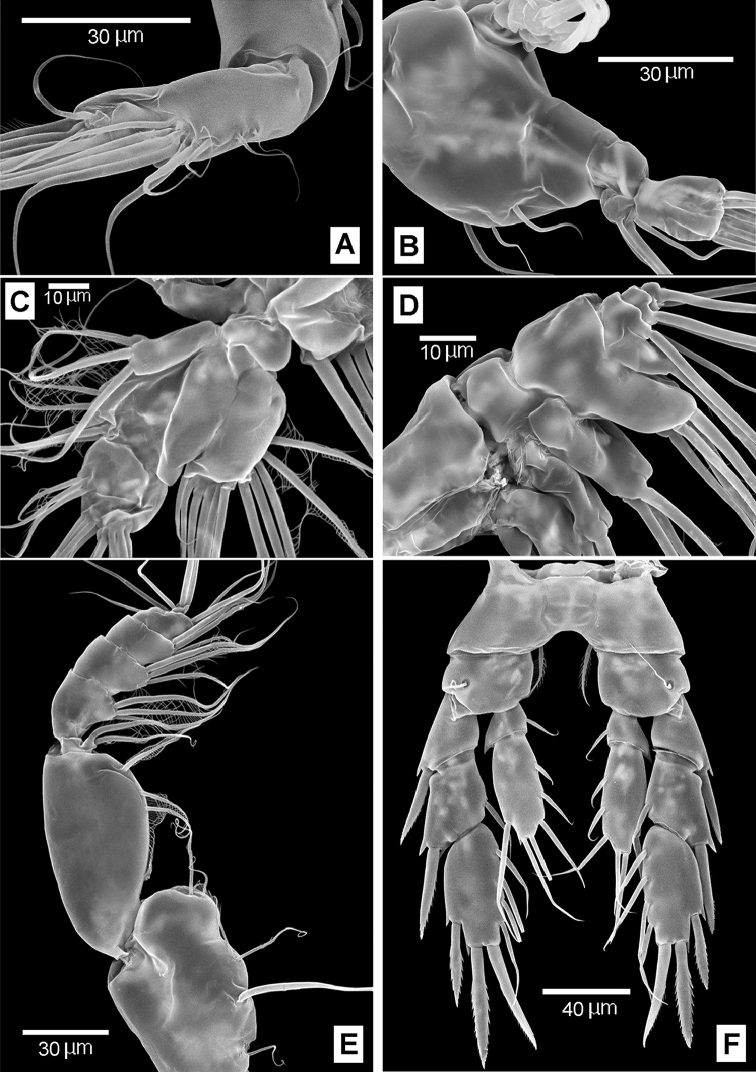
*Boholina reducta* sp. nov., SEM micrographs, female. **A** distal segment of antennary endopod **B** mandibular palp **C** maxillula **D** maxilla **E** maxilliped **F** P5, posterior.

Antennae, mouthparts and P1–P4 as in female.

**Figure 9.**
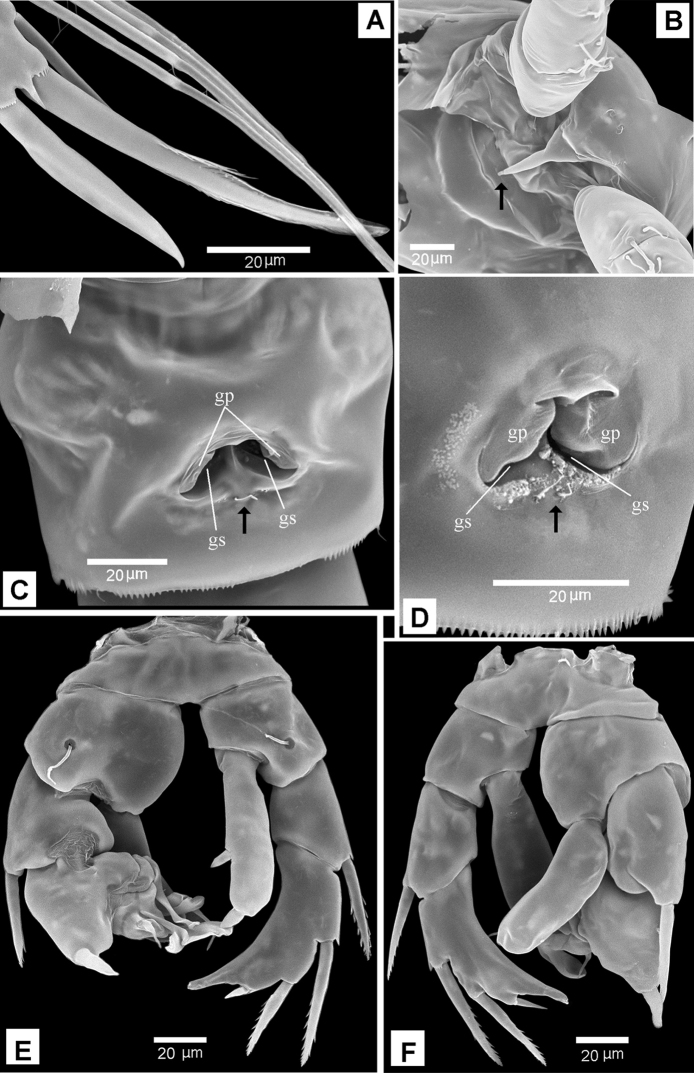
*Boholina reducta* sp. nov., SEM micrographs **A–D** female **E–F** male **A** distal spines of P4 Exp-3 **B** rostrum, ventral view (arrow indicating tip of rostrum) **C** genital double-somite, ventral view (arrow indicating sensilla near gonopores) **D** gonopores, latero-ventral view (arrow indicating sensilla near gonopores) **E** P5, posterior **F** P5, anterior. Abbreviations: gp, gonoporal plates; gs, gonoporal slits.

P5 (Figs 7B, C, 9E, F) strongly asymmetrical, biramous; coxae and intercoxal sclerite fused to form common base, without armed elements on anterior and posterior surface. Right P5: basis about as long as wide, with slender outer basal seta located on posterior surfaces; exopod 2-segmented, first segment with long bilaterally serrate outer spine (39–42 µm), distal segment large in base and tapering on the tip, slightly curved inward, inner margin smooth; outer margin armed with three spines, proximal serrated spine (48–51 µm), middle serrated spine (33–38 µm) and distal short spine vestige (9–11 µm), terminal spine absent; endopod forming an elongate lobe, about 3.8 times as long as wide, armed with two slightly sigmoid spines, apical spine 11–13 µm long and inner spine 8–9 µm, subdistally. Left P5: basis robust, about 1.08 times as wide as long, with slender outer basal seta located on posterior surface; exopod 3-segmented, ﬁrst segment with a long serrate outer spine (40–42 µm), second segment modiﬁed, bearing strongly reﬂexed spine (35–38 µm) on outer margin; third segment highly transformed bearing multiple short processes and one long, naked modified seta; endopod unarmed, forming an elongate rounded lobe, about 3.2 times as long as wide.

##### Variability.

One female paratype (IEBR-COP3488) and three females among ten additional specimens examined showed asymmetrical P5 Exp-3, with a distal inner seta on right leg (Fig. 5E, arrows) while lacking on left one (Fig. 5F, arrow).

##### Remarks.

The new species agrees well with the generic diagnosis of *Boholina* given by Fosshagen and Iliffe (1989), Boxshall and Jaume (2012), Moon and Soh (2014), and Boonyanusith et al. (2020): fourth and fifth pedigerous somites completely fused; urosome 4-segmented in the female and 5-segmented in the male with very short anal somite, telescoped within the preceding free abdominal somites in both sexes; genital openings paired, located ventromedially or ventrolaterally of genital double-somite; caudal rami produced into a pointed dorsal process in the middle of the distal margin; female antennule 24-segmented, with segments 8 and 9 partly fused or completely separated; P1 with 3-segmented endopod, each segment with a pointed outer distal corner, distal segment without any outer seta; P4 with slightly modified distomedial spine on the distal segment of the exopod; P5 with 2-segmented endopod in the female; and in the male P5 with a complex grasping organ and a highly modified exopod, reduced 1-segmented endopod on both sides.

Among six congeneric species currently recognized in *Boholina*, *B. reducta* sp. nov. shares the paired gonopores located either side of the ventral midline with *B. ganghwaensis*, *B. parapurgata* and *B. purgata*, and shares rounded postero-lateral corners of the second and third free pedigerous somites with *B. munaensis*, *B. crassicephala* and *B. laorsriae*. The new species is similar to *B. laorsriae* by the medial lobe of the distal segment of the antennary endopod having nine setae (while other congeners have eight setae). *Boholina reducta* sp. nov. resembles *B. munaensis* in bearing the single seta on the outer margin of the female P5 Endp-2 (against two in the other congeners). The new species is also similar to *B. ganghwaensis* in having the distal segment of the mandibular palp endopod with 11 setae (versus ten setae in the other congeners) (Table 2).

**Table 2.** Morphological comparison of seven species of the genus *Boholina*.

	*B. ganghwaensis*	*B. parapurgata*	*B. purgata*	*B. munaensis*	*B. crassicephala*	*B. laorsriae*	*B. reducta* sp. nov.
***Female***							
Body length (mm)	1.03–1.29	0.93–1.11	0.73–0.79	0.70–0.77	0.75–0.85	0.68–0.73	0.86–0.94
Posterior angle of tergites of pedigerous somites 2–3	Pointed	Pointed	Pointed	Rounded	Rounded	Rounded	Rounded
Shape of rostrum	Narrow rounded	Narrow rounded	Narrow rounded	Transverse crest	Transverse crest	Narrow rounded	Narrow pointed
Gonopores on double-somite	Either side of ventral midline	Either side of ventral midline	Either side of ventral midline	Ventrolaterally	Ventrolaterally	Ventrolaterally	Close together on ventral midline
Small hook-like inner process on gonoporal plate	Present	Absent	Absent	Absent	Absent	Absent	Absent
Septum between gonopores	Narrow	Narrow	Narrow	Large	Large	Large	Reduced
Length/width ratio of caudal ramus	1.6	1.5	?	1.5	?	1,8	1.5
Distal exopodal segments of antenna	Completely separated	Completely separated	Not separated?	Completely separated	Not separated?	Completely separated	Completely separated
No. of setae on medial lobe of antennary Enpd-2	8	8	8	8	8	9	9
No. of setae on basis of mandibular palp	4	4	4	4	4	4	3
No. of setae on distal endopodal segment of mandibular palp	11	10	10	10	10	10	11
Setal formula on exopod of mandibular palp	1:1:1:1:2	0:1:1:1:2	1:1:1:1:2	0:1:1:1:2	1:1:1:1:2	1:1:1:1:2	1:1:1:1:2
Basis and ﬁrst endopodal segment of maxillule	Incompletely separated	Fused	Fused	Fused	Fused	Separated	Incompletely separated
First and second endopod segment of maxillule	Incompletely separated	Separated	Fused	Separated	Fused	Fused	Fused
No. of setae on exopod of maxillule	10	10	10	10	10	10	11
Seta on basal exite of maxillule	Present	Present	Present	Present	Present	Present	Absent
Setal formula on endopod of maxilliped	2:4:4:3:3+1:4	2:4:4:3:3+1:4	2:4:4:3:3+1:4	2:4:4:3:3+1:4	2:4:4:3:3+1:4	2:4:4:3:3+1:4	2:3:3:3:3+1:4
Outer seta on P3 basis	Present	Absent	Present	Absent	Present	Present	Present
Location of spinule row of distomedial spine of P4 Exp-3	Distal end	Distal end	Distal end	Distal end	Distal end	Distal end	Middle
Length/width ratio of distal endopod segment of P5	2.5	2.6	?	2.6	?	2.0	2.4
Length ratio of inner distal spine and outer terminal spine of P5	1.03	0.78–0.82	?	1.40	?	1.8	1.0
No. of spines on outer margin of P5 Exp-3	2	2	2	2	2	2	1
No. of setae on inner margin of right P5 Exp-3	3	3	3	3	3	3	4
No. of setae on outer margin of P5 Endp-2	2	2	2	1	2	2	1
***Male***							
Body length (mm)	0.87–0.93	0.66–0.71	0.64–0.73	0.68	0.70–0.77	0.65–0.67	0.76–0.83
Process at the antepenultimate segment of right antennule	Present	Absent	Present	Absent	Present	Absent	Absent
Apical spine on P5 basis	Present on both legs	Present on right leg only	Present on right leg only	Absent	Absent	Absent	Absent
No. of segments of right P5 exopod	1	1	1	1	1	1	2
Armature on the distal segment of right P5 exopod	3 strong + 1 vestigial spines	3 strong + 1 vestigial spines	3 strong + 1 vestigial spines	4 strong spines	4 strong spines	4 strong spines + 1 short spiniform seta	2 strong + 1 vestigial spines
Length/width ratio of right P5 endopod	3.2	3.6	2.6	?	?	3.0	3.8
Large inner spiniform process on right P5 endopod	Absent	Absent	Absent	Present	Absent	Absent	Absent
Armature of right P5 endopod	2 slender spines	2 sigmoid spines	2 slender spines	Absent	2 slender spines	2 slender spines	2 sigmoid spines

However, *B. reducta* sp. nov. is distinguished from all six congeners by the unique characteristics as follows (see Table 2): (1) a pair of gonopores are located close together on the mid-ventral surface of the genital double somites, and the septum between gonopores is only visible in the inner part of the genital opening. In *Boholina*, there are three species (*B. ganghwaensis*, *B. parapurgata* and *B. purgata*) with gonopores located on either side of the ventral midline on genital double somites. However, the separation between gonopores is clearly visible in ventral view of the genital double somites (Fosshagen and Iliffe 1989; Boxshall and Jaume 2012; Moon and Soh 2014). In other species of *Boholina*, the gonopores are widely separated (Fosshagen and Iliffe 1989, Boonyanusith et al. 2020), (2) the rostrum has a narrow finger-like process with pointed tip. The shape of rostrum of new species is unique in the Pseudocyclopidae, (3) the basis of mandibular palp has three setae in the new species, while there are four setae in all the species of *Boholina*, (4) there is no outer seta on basal exite of maxillule and there are 11 setae on exopod of maxillule, while there are only ten setae in other species of *Boholina*, (5) the second and third endopodal segments of maxilliped have three setae each, (6) the distomedial spine of P4 Exp-3 is modified with a row of spinules inserted in the middle of inner margin of the spine, (7) the female P5 Exp-3 has only one spine on outer margin, and the proximal outer spine is missing. In *Boholina*, the outer margin of female P5 Exp-3 generally has two spines, (8) female P5 Exp-3 has four setae on the inner margin, while there are three setae both rami in the other species of *Boholina* (Fosshagen and Iliffe 1989; Boxshall and Jaume 2012; Moon and Soh 2014 and Boonyanusith et al. 2020), (9) right P5 exopod has two segments in the male, and (10) the distal segment of right P5 exopod in male has only two strong spines and one short vestigial spine on outer margin and the terminal spine of the segment, which are unique in the genus. This is the first record of *Boholina* and Pseudocyclopidae from Vietnam waters. An updated key to the seven valid species of *Boholina* is provided.

### A key to species of the genus *Boholina* (modified from Boonyanusith et al. 2020)

**Table:** 

1	Female P5 Exp-3 with four spines in total; male right P5 exopod 1-segmented and distal segment with terminal spine	**2**
–	Female P5 Exp-3 with three spines in total; male right P5 exopod 2-segmented and distal segment without terminal spine	***Boholina reducta* sp. nov.**
2	Gonopores in female located ventrolaterally; male right P5 exopod with four well-developed spines	**3**
–	Gonopores in female located close together on mid-ventral surface; male right P5 exopod with three well-developed spines	**5**
3	Female P5 Endp-2 with one seta on outer margin; male right P5 endopod with large spinous process on inner margin	***B. munaensis* Boxshall & Jaume, 2012**
–	Female P5 Endp-2 with two setae on outer margin; male right P5 endopod without large spinous process on inner margin	**4**
4	Inner apical spine on female P5 Exp-3 about 1.8 times as long as outer one; male right P5 exopod with minute spiniform seta on inner margin; male left P5 endopod small, much shorter than right P5 endopod	***B. laorsriae* Boonyanusith, Wongkamhaeng & Athibai, 202**0
–	Apical spines on female P5 Exp-3 subequal in length; male right P5 exopod without spiniform seta on inner margin; male left P5 endopod large, as long as right P5 endopod	***B. crassicephala* Fosshagen & Iliffe, 1989**
5	Two apical spines on female P5 Exp-3 shorter than segment; the antepenultimate segment of male right antennule with rounded process; male right P5 endopod with two slender spines	**6**
–	Outer terminal spine on female P5 Exp-3 longer than segment, inner apical spine just shorter; the antepenultimate segment of male right antennule without process; male right P5 endopod with two sigmoid spines	***B. parapurgata* Boxshall & Jaume, 2012**
6	Outer terminal spine on female P5 Exp-3 shorter than inner apical spine; female gonoporal plate with small hook-like process; male right P5 endopod about 3.2 times as long as wide	***B. ganghwaensis* Moon & Soh, 2014**
–	Outer terminal spine on female P5 Exp-3 longer than inner apical spine; female gonoporal plate without small hook-like process; male right P5 endopod about 2.6 times as long as wide	***B. purgata* Fosshagen & Iliffe, 1989**

## Supplementary Material

XML Treatment for Boholina reducta
